# The Cultural Formulation Interview as a clinical tool in the assessment of eating disorders: a pilot study

**DOI:** 10.3389/fpsyt.2024.1371339

**Published:** 2024-04-12

**Authors:** Mattias Strand, Elisabeth Welch, Sofie Bäärnhielm

**Affiliations:** ^1^Centre for Psychiatry Research, Department of Clinical Neuroscience, Karolinska Institutet, Stockholm, Sweden; ^2^Transcultural Centre, Northern Stockholm Psychiatry, Stockholm Health Care Services, Region Stockholm, Stockholm, Sweden; ^3^Global Health and Migration Unit, Department of Women’s and Children’s Health, Uppsala University, Uppsala, Sweden; ^4^Division of Psychology, Department of Clinical Neuroscience, Karolinska Institutet, Stockholm, Sweden

**Keywords:** anorexia nervosa, bulimia nervosa, binge eating, culture, migration, perfectionism, religion, racism

## Abstract

**Background:**

The Cultural Formulation Interview (CFI) in the DSM-5 is a person-centered instrument for systematically appraising the impact of sociocultural factors in psychiatric assessment. The CFI has been shown to be feasible, acceptable, and useful in various clinical contexts. However, to this date there is only one published report describing the use of the CFI with patients with eating disorders.

**Aims:**

To explore the potential benefits and challenges of utilizing the CFI in the assessment of eating disorders.

**Methods:**

As an addendum to an ongoing qualitative study about barriers to treatment for eating disorders for individuals with a migration background in Sweden, we utilized the CFI in the assessment of adult patients (n=8) in specialist eating disorder treatment. Interview data were analyzed employing a thematic analysis framework. Participants provided feedback using a standard form for evaluation of the CFI.

**Results:**

Certain CFI questions proved especially meaningful in this context. In response to the CFI question about patient explanatory models, previously unrecognized ideas about causation emerged. These included perfectionism—a known risk factor for eating disorders—based on immigrant parents’ career expectations and experiences of strict family control over life choices. In response to the CFI questions on cultural identity and its impact, the participants provided rich descriptions including important themes such as religion, racism, and ambiguities associated with being a second-generation immigrant. The final CFI question, eliciting concern about the patient-clinician relationship, revealed numerous examples of prejudice and unfamiliarity with migrant groups among healthcare providers.

**Implications:**

The CFI can be useful in the assessment of patients with eating disorders and should be further explored as a standard tool in specialist eating disorder services.

## 1 Introduction

Eating disorders such as anorexia nervosa, bulimia nervosa, or binge eating disorder used to be seen as conditions that mostly afflict White women from socioeconomically privileged backgrounds (1), to the extent that, occasionally, eating disorders have been described as a uniquely Western “culture-bound syndrome” (2). More recently, this stereotypical view has been challenged by research findings demonstrating that eating disorders affect people from all parts of society, regardless of gender, ethnicity, or socioeconomic background (3). Eating disorders give rise to a substantial global burden of disease (4, 5) and are becoming more common worldwide (6), not least in East and Southeast Asia (7). Unfortunately, there is little research on the impact of migration on the development of eating disorders and on treatment seeking. Need for such research is evident from a recent Swedish study, which showed that disordered eating is substantially more common among individuals born abroad—especially so among migrants from Asia and Africa—compared to the Swedish-born population (8). The same was found to hold true for individuals with parents born in another country (the so-called “second generation”) and individuals residing in a neighborhood with a large proportion of migrant residents. Even so, individuals with a migration background are clearly underrepresented in Swedish specialist eating disorder treatment; a pattern that is also observed in other parts of the Global North and that has been described as an “eating disorders blind spot” (9).

Sociocultural differences in how patients and clinicians think about and make sense of health and illness can contribute in raising barriers to effective communication, assessment, and treatment planning in the clinical setting. The cultural components that synergistically shape our mental models—such as gender, age, ethnicity, language, socioeconomic status, educational background, and religious beliefs—may also hinder insight into the lived experiences of others, give rise to misunderstanding, and negatively affect the establishment of rapport and trust. Failing to recognize and address such barriers can ultimately result in poor treatment adherence, negative outcomes, and prolonged illness. The Cultural Formulation Interview (CFI), included in the fifth edition of the *Diagnostic and Statistical Manual of Mental Disorders* (DSM-5), is a person-centered instrument for systematical appraisal of the impact of cultural factors on the clinical encounter (10). The core component of the CFI comprises 16 open-ended questions related to the respondent’s cultural understanding of health and illness; sociocultural identity, stressors, and resources; cultural aspects of coping and help-seeking; and the patient-clinician relationship. There are also 12 supplementary modules that can be used to address topics relevant for specific populations, such as children and adolescents, the elderly, or migrants and refugees. The CFI has proven to be a feasible, acceptable, and useful tool for exploring cultural context and identifying treatment barriers in various clinical settings (11–13). For example, in the field trials preceding the inclusion of the CFI in the DSM-5—including 318 patients and 75 clinicians over 11 sites in six countries—patient ratings of feasibility, acceptability, and clinical utility were positive, with mean overall results for all three outcomes scoring 1.26–1.33 on a scale ranging from -2 to 2 (11). However, the potential role of the CFI in the assessment of eating disorders is currently poorly understood.

To the best of our knowledge there is only one published research article reporting on the use of the CFI in patients with an eating disorder: an Israeli case report describing two Israeli-Ethiopian adolescent girls presenting with restrictive eating (14). In this study, the CFI was found useful in elucidating certain cultural aspects of the patients’ symptomatology, such as a notion of the stomach—rather than the heart or the head—as an epicenter of emotions in Ethiopian folk psychology.

The present study was designed as an addendum to an ongoing qualitative study about barriers to treatment for eating disorders for individuals with a migration background in Sweden. The aim of the study was to explore the potential benefits and challenges of utilizing the CFI in the assessment of eating disorders in this patient group. More specifically, the study objectives were:

a) To examine whether using the CFI in a specialist eating disorder treatment setting would contribute useful information that had not previously been addressed.
b) To explore if any of the CFI questions would prove to be particularly difficult to understand and/or answer for the participants.

Furthermore, we had also hoped to be able to formally assess the participants’ views on feasibility and acceptability of the CFI, utilizing a standard research instrument for evaluation. However, due to the relatively small number of participants, no firm conclusions in this regard can be drawn. We nevertheless provide a summary of these quantitative data as an indication of how our participants experienced the CFI.

## 2 Materials and methods

### 2.1 Participants

To be eligible for inclusion in the study, participants had to be ≥18 years of age, have a history of being treated for an eating disorder, and self-identify as having a migration background, as belonging to a minority ethnic group, or as being a person of color. Participants were recruited with the help of advertisements in various formats in receptions and waiting rooms at the outpatient, day treatment, and inpatient wards of the participating specialist services. Patients enrolled in Internet-based treatment were also informed about the study and invited to participate. Furthermore, clinicians were encouraged to inform eligible patients about the study and ask them if they were interested in participation. The stated purpose of the study was twofold: i) to explore barriers to treatment for eating disorders for individuals with a migration background in Sweden in an open-format semi-structured interview, and ii) to explore the potential benefits and challenges of utilizing the CFI in the assessment of eating disorders in the same group. Participants could choose to partake in either or both interviews; all chose to partake in both. The present article reports the findings from the CFI part of the study. Interviews were conducted with eight adult participants (seven women and one man; mean age: 35 years, range: 18-46 years) who were currently in treatment, waiting to resume treatment, or who had at the time of the interview recently ended their treatment contact at one of the government-run specialist eating disorder services, either in Stockholm or Gothenburg. In terms of migration background, one participant had immigrated to Sweden as an adult, three had immigrated as children with their families (sometimes referred to as the “1.5 generation”), two were born in Sweden to immigrant parents, and two were transnational adoptees who had come to Sweden as young children. The participants and/or their parents had migrated from seven different countries in Sub-Saharan Africa, the Middle East, South or East Asia, the former Soviet republics, and South America.

### 2.2 Procedures

Using the authorized Swedish translation of the CFI (15), individual interviews lasting between 25 and 50 minutes were performed. A real-life clinical approach was taken with regards to instrument fidelity, allowing for rephrasing of the questions according to the individual participant’s style of communication without altering the intended meaning and purpose. All interviews were digitally recorded and transcribed verbatim. The transcripts were pseudonymized by omitting or altering potentially identifying details, such as names, country of birth, and occupation.

The CFI Debriefing Instrument for Patients (11), which has previously been translated to Swedish and slightly adapted (16), was used to evaluate the participants’ perceptions of clinical utility, feasibility, and acceptability.

### 2.3 Analysis

The transcribed interview data were analyzed employing what is sometimes called a codebook thematic analysis approach (17), combining the methodological framework outlined by Braun and Clarke (18) with elements of conventional qualitative content analysis (19). In choosing this approach, the aim of the analysis was to describe important themes elicited by the CFI questions rather than to theorize. In an iterative process, the data was first read and re-read and initial ideas for coding categories and overarching themes were drafted. Second, the transcripts were coded and labelled according to a ‘bottom-up’ principle, avoiding preconceived ideas about topics that might prove to be meaningful in the participant narratives. Third, the categories were grouped into themes. Due to the highly structured nature of the CFI, themes were not identified across the interview questions; instead, we chose to use the 16 CFI questions as an overarching thematic framework and to identify meaningful themes within each question. In doing this, we focused on identifying those questions that elicited information of potential relevance to assessment and treatment. No predefined criteria (such as a specific number of participant statements needed) were applied in determining what would constitute a separate theme; instead, meaningful clusters were identified and developed inductively by analyzing patterns in the data elicited by each CFI question. Fourth, the themes were reviewed and reworked using mapping techniques in order to achieve an optimal structure for describing the data. Finally, illustrative pseudonymized quotes were chosen and the findings were presented and contextualized.

### 2.4 Reflexivity

During the interview phase of the study, MS (the author conducting the interviews) continuously reflected on his own position vis-à-vis the participants, the study context, and the research questions. These reflections were recorded in the form of “field notes”. Importantly, a form of instant reflexivity is also built into the CFI, since the last CFI question specifically addresses potential misunderstandings arising from differences between the sociocultural background of the interviewer and that of the interviewee. An obvious aspect that may have affected the interview situation is MS’s role as White male clinician-researcher interviewing patient-participants mostly identifying as persons of color about topics related to cultural identity, migration, “in-betweenness”, and racism. When this dynamic was explicitly addressed, participants would typically acknowledge that it might potentially affect their willingness and ability to talk openly on these subjects, but that the study setting and the non-rigid nature of the interview procedure helped in establishing a situation in which they could feel safe and reasonably comfortable. Some of the participants specifically mentioned that they had chosen to contribute to the study because they saw the study aims as important and personally meaningful; this would also help to create an open atmosphere during the interviews. Also, MS has previously worked as a clinician at one of the specialist services where interviews were conducted. However, only one of the participants had been in treatment long enough to recognize this and it is unlikely to have affected the interview. None of the authors were involved in the treatment of any of the participants at the time of the study.

### 2.5 Ethics and preregistration

This study was conducted in accordance with the ethical standards of the Helsinki Declaration of 1975, as revised in 2008. The study was approved by the Swedish Ethical Review Authority (Nos. 2021-05935-01, 2022-06457-02, and 2023-03817-02). Written consent was obtained from all participants. The study protocol has been preregistered on the Open Science Framework (osf.io/acfdy).

## 3 Results

A schematic summary of the themes identified for each of the 16 CFI questions are shown in **Table 1**. Some of the CFI questions clearly contributed more than others in elucidating aspects of potential relevance to the assessment and treatment of the participants; these questions are marked with a “✓” in the table and discussed in more detail below.

**Table 1:** Cultural Formulation Interview questions and themes.

	CFI question^†^	Themes	Notes	
1	Describing the problem	• Clinical description of an eating disorder		
2	Describing the problem to family or friends	• Clinical description of an eating disorder	Important for participants to “own” the diagnosis, but relatives would not necessarily understand	✓
3	Identifying the most troubling aspects	No distinct patterns		
4	Exploring ideas about causation	• Perfectionism • Lack of control • Sociocultural meaning of food	Sociocultural meaning of food only mentioned by a few, but proves important for more when specifically asked for	✓
5	Exploring ideas about causation among family or friends	• Immaturity	May be used to take away control (see question 4): “If you can’t even eat, you’re not mature enough to make decisions about your life”	✓
6	Identifying supportive factors	No distinct patterns	Participants describe a general lack of support	
7	Identfying stressors	• Economy • Unhealthy body ideals among relatives	For example, relatives constantly engage in body talk and exhibit disordered eating behaviors, but do not label this as an eating disorder	✓
8	Discussing cultural background and cultural identity	• Religion • “In-betweenness” • Experiences of racism • Professional identity	Most participants initially experience the question as difficult, but they can then readily go on to provide rich and nuanced answers	✓
9	Impact of cultural identity on the current problem	• Stigma among other migrants • Finding a partner	For example, expectations of not engaging in recreational premarital dating means that you need to always “look your best” in order to attract “the right one” when the time is right	✓
10	Other problematic impact of cultural identity	No distinct patterns		
11	Identifying coping mechanisms	No distinct patterns		
12	Discussing past help seeking	No distinct patterns		
13	Identifying barriers to help seeking	• Economy (same as question 7)	Many other relevant barriers identified in a separate longer interview, but not here	
14	Discussing preferences for help	No distinct patterns		
15	Discussing suggestions from family or friends	• Religion • Visit country of origin • Usual “dad wisdom”	Typical “helpful” comments from relatives, such as “you just need to eat”, “have a banana!”, etc. that most patients with an eating disorder can report	✓
16	Addressing misunderstandings in the clinical encounter	• Discrimination • Downplaying racism • Pressure from non-White therapists • Lack of insight into other cultures • Pressure to break with family	This question did not elicit many accounts of misunderstandings during the present interview, but clearly prompted participants to discuss previous experiences	✓

^†^These brief descriptions of the CFI questions are merely labels; they are not meant as definitive accounts of the content and purpose of the questions. For full questions and additional information for interviewers, please refer to American Psychiatric Association 2013. Questions that proved particularly useful for eliciting information of potential relevance to assessment and treatment in this context are marked with an “✓”.

### 3.1 Question 2: describing the problem to family and friends

#### 3.1.1 Clinical description of an eating disorder

Most participants stated that they would use formal diagnostic terminology or a clinical description of an eating disorder when describing their problem to family and friends, similar to how they would describe it to a healthcare provider. Some of them even made a point of being frank about their problem and not letting shame or stigma prevent them. However, many were uncertain whether people in their community would actually understand what they meant:

*“I don’t think my family members even know what an eating disorder is. I couldn’t say that. I wouldn’t know what to say. Repetitive vomiting? But that would also be difficult to understand.”*

### 3.2 Question 4: exploring ideas about causation

#### 3.2.1 Perfectionism

A recurring theme in participants’ ideas about what had contributed in causing an eating disorder was perfectionism:

*“Early in life there was this hyper-focus on achievements and being good and not making mistakes. And to perform academically. And then, for me, food became a weakness. Even though I know intellectually that you need food because you need energy and fuel in order to perform even better.”*

Notably, this was often discussed in relation to parental expectations. Several participants explained how knowing that their parents had gone through hardships in order to be able to migrate and create a new life for themselves in Sweden made them feel a pressure—explicit or implicit—to succeed and make the parents’ struggle worthwhile:

*“To become a doctor, something high-status, an engineer. Everything is about that. Not that I really know what I want to do, but they’re still pushing me in that direction.”*

This feeling would also sometimes be coupled with experiences of having to work harder than their non-migrant peers in order to succeed in school or at work, due to lack of support or discrimination and racism. Furthermore, some participants would refer to culture in a more general sense:

*“Early in life there was this hyper-focus on achievements and being good and not making mistakes. And to perform academically. And then, for me, food became a weakness. Even though I know intellectually that you need food because you need energy and fuel in order to perform even better.”*

*“Everything needs to be perfect. Perhaps that’s also a cultural thing, that you need to be a perfect mother, a perfect colleague, a perfect friend, a perfect girlfriend. And look good and have a nice clean home. No bad habits. A good salary.”*

There were also descriptions of ‘high-performance culture’ at work or in peer groups contributing to feelings of inadequacy:

*“It was the people I hung out with. For example, when I was younger I had … All my friends would have a minimum of two master’s degrees. Everybody went to [Swedish top university].”*

#### 3.2.2 Lack of control

Several participants described feelings of lack of control in different areas of life as a contributing factor, turning food and eating in to an arena where a sense of rigid control could be regained. The perceived lack of control would often involve real-life experiences of relatives being in charge of important choices about their future:

*“This culture of honor defines my life. Life choices, how I want to live my life. If I’d like to move away [to study]—no, I can’t move. Because a girl who isn’t married can’t move. It’s probably not shameful, but it doesn’t look good.”*

One participant also mentioned how this aspect had made her less motivated to seek treatment and try to get better:

*“There’s this thought that I’m still not going to be in charge of my life. Why am I even doing any of this? [ … ] This tiny bit of control that I have over my life. Compared to the control my family has over me.”*

#### 3.2.3 Sociocultural meaning of food

There were very few mentions of sociocultural aspects of food and eating in the participant responses. Only one participant brought this up, describing how her parents’ and grandparents’ experiences of growing up in poverty and starvation gave food a special significance in her family that she now felt contributed to tendencies of binge eating:

*“Food was something that brought everybody together and you couldn’t say no. There was always lots of food on the table when we ate.”*

### 3.3 Question 5: exploring ideas about causation among family or friends

#### 3.3.1 Immaturity

Several participants mentioned that they had never before reflected upon what their relatives might see as the cause behind their problem. A recurring theme was experiences of being seen as an immature adolescent, even though all participants were adults. Notably, this would sometimes be used as an argument for family control of over important life choices as described above:

*“Many times, my family and other people in my life have used my vulnerability to control me. You know: ‘Well, you obviously can’t handle this yourself, so I’ll tell you what choices to make instead.’”*

### 3.4 Question 7: identifying stressors

#### 3.4.1 Economy

Financial matters were often perceived as a stressor. Interestingly, many participants would spontaneously discuss this as a barrier to help seeking (forestalling question 13). Lack of money could, for example, prevent participants from improving their eating behaviors:

*“As long as I’m home, I eat or drink strained tomatoes, I eat salad. It would have been nice with more fruit, but we can’t afford it. It’s like … The variety of foods—fish, for example, that I’d like to eat, that I know is good for me—it’s just not an option. We can’t afford it.”*

Others mentioned concern for the family’s economy as a reason for not involving parents in treatment:

*“Not the eating disorder as such, but being in treatment, as I said. I thought a lot about how to get better without affecting my parents’ jobs and their economy.”*

#### 3.4.2 Unhealthy body ideals among relatives

A couple of participants mentioned that relatives in their country of origin were constantly engaged in body talk and exhibited disordered eating, although the relatives did not label this as an eating disorder:

*“My parents are very body conscious. [ … ] So there has been a lot of focus on food and on the body. Both my parents aim to lose weight. It’s very important.”*

### 3.5 Question 8: discussing cultural background and cultural identity

All participants experienced the question about cultural background and cultural identity as difficult. However, after hearing it twice or having briefly discussed what was meant by these terms (including, for instance, reiterating the examples of aspects of cultural identity given in the question), they could all produce rich and nuanced accounts.

#### 3.5.1 Religion

A recurring theme when discussing important elements of the participants’ cultural identity was religion and spirituality:

*“I was raised Catholic. But I’m not so … [ … ] What’s important to me is faith. To believe in something. It has helped me several times when I’ve felt that ‘God, I’m losing it’. I’ll call upon the Universe. I have Indian background too—both in the United States and in Venezuela. I mean, both Wayuu and the Indians there. In my family, we often talk about Mother Earth and all that, from my dad’s side. Which makes it my belief too.”*

Many participants specifically mentioned that their own religiosity had been molded in dialogue with and as a protest against the stricter religious views of the parental generation:

*“I’m not super big on religion, but I do have a secure faith in God. I’ve had a hard time understanding religion. When I was little, it was so associated with cultural things, which faith isn’t for me. It was like: ‘God will punish you if you do this, God doesn’t like it when you do that.’ And I just: ‘But what kind of God is this, why should I believe in this God? It’s no good.’ But since I’ve gotten older, I found a faith in God beyond religion. And that’s what I think is so nice about religion, that you can search within and see what you feel and believe in.”*

Importantly, all participants that brought up religion as a vital aspect also said that this theme had not been addressed by their therapists, even though their religious faith was clearly a source of support in times of distress.

#### 3.5.2 “In-betweenness”

A sense of in-betweenness associated with belonging to multiple cultural spheres (“mellanförskap” in Swedish) was mentioned by most participants. Those who had grown up in Sweden as children to immigrant parents described how they had constantly had to navigate liminal spaces and negotiate belonging. For some, this was mostly seen as positive; as a mobility of the self that allowed them to tap into resources from different spheres:

*“Because I am Swedish, I feel Swedish, I think Swedish, whatever that is. But I also care a lot about my family and I respect them. So it’s like: I want to live my own life, but I also want to do good for my family. It’s like being in-between.”*

*“I suppose that if I had been raised in Malaysia, I would be more religious than how it is now. But I’m Swedish, I grew up in Sweden, I’ve gone to a Swedish school and everything, and my values are Swedish. With elements of … I think that religion, for me, is more about deep values, sort of like how you treat others and what kind of person you are—being good, being generous. But being Swedish is more about how you view society, how you think about what’s ok and not.”*

However, this state of in-betweenness could also be experienced as a barrier to inclusion or as demanding and conflicted:

*“I understand why my parents … I understand why they chose to live in the heart of the city, why they chose those schools, I understand it intellectually but I feel like I’ve missed a huge part of my culture and that I’ve missed the natural community I would have had if we had lived where other Nigerians live. So I’ve always felt this in-betweenness.”*

*“You have to live somewhat of a double life when you have immigrant parents. And I have a lot of relatives who really live double lives. I mean, they’re somebody completely different with their parents compared to when they’re out of the house. [ … ] I accept their rules for the time being. But then when I have my own life, they can’t tell me to change.”*

#### 3.5.3 Experiences of racism

Many participants mentioned experiences of racism and othering as integral part of their cultural identity:

*“Where I grew up, it was like this: If I opened my mouth and said that I was adopted, I got an OK stamp. Then I was excused, looking like I do. If I didn’t, I was a f-ing immigrant and you didn’t want that. The human hierarchies were so sharp. So I learned to say, quick as hell: ‘Hi, I’m Cecilia and I’m adopted.’ So twisted. One long apology: ‘Sorry that I look like this, but I’m like you. I also eat meatballs with lingonberries.’”*

Some of them would play down these experiences as not being such a big deal, but they were nevertheless mentioned as an element of cultural identity:

*“I’ve heard some ‘ching chong’ stuff. I have, but not that much.”*

Similar to the theme of religiosity, those who described experiences of racism as important said that this had not been addressed by their therapists.

#### 3.5.4 Professional identity

Several of the participants mentioned their professional identity as an important facet of cultural identity, sometimes for lack of other sources of pride and self-confidence:

*“My job is really the only thing in my life that’s been sort of my guiding light. Something that nobody can take from me. That I created myself, that I can control, that nobody else can … [ … ] At work, I can let go of regular Isaac and be Isaac the professional. I have a framework, I know what’s expected of me, I know what to do, and I don’t need to think about anything else.”*

*“I’ve never felt like I was enough of anything, in any context. But perhaps that’s why being a psychologist became extra important to me, because that role was mine, I had fought for it myself. And I think that’s why the crisis hit me so bad last year. Because I felt like I didn’t know who I was if I couldn’t work. So I think my job has always been a huge part of my identity. Unfortunately.”*

This theme was also clearly linked to the tendency for perfectionism described above; discussing professional identity, one participant even asked herself:

*“Could always performing well be one’s cultural identity?”*

### 3.6 Question 9: impact of cultural identity on the current problem

#### 3.6.1 Stigma among other migrants

Lack of knowledge about and stigmatization of mental disorders were mentioned by several participants:

*“It’s about my heritage, that I’m from the Congo. Spending time with my fellow countrymen and keeping up the … When there are get-togethers, cultural festivals, and different events—that just came to an end because I’ve felt bad. And you don’t get the same kind of understanding. Mental distress doesn’t exist in that sphere. For them it’s … If you’re bleeding or you break a bone, then you feel bad. That’s illness.”*

*“My parents would probably have preferred it if nobody knew that I have an eating disorder, at least. I think the first step for them was: ‘Do you really need to see a psychiatrist?’ and yada yada yada. But the next step is: ‘Ok, do what you got to do, but must everybody really know it?’ So I’m currently working on overcoming those feelings of shame.”*

For some, experiences of ‘fat stigma’ was specifically associated with being a part of a migrant community:

*“I have three Swedish friends that I’ve known for a long time, 15 to 20 years. But my other 20 friends are Russian speakers from different countries. And then there’s this expectation that you should be thin and pretty and perfect in every way. [ … ] You should always be perfect, never be weak, always look pretty. It’s a woman’s responsibility to look good all the time. And looking good also means having a perfect body. If not, you’re half a woman or something.”*

#### 3.6.2 Finding a partner

A couple of participants mentioned how being affected by honor-based family culture meant that appearance and body consciousness carried a special significance for them, based on the notion that they did not have many shots at finding a partner:

*“It’s a way of thinking that my parents taught me, that you can’t date, you can’t have more than one boyfriend and the one you meet is who you should be with for the rest of your life. And then I felt: ‘Ok, how am I going to find Mr Right, the potential partner that I want and that actually wants me back?’ Then I got to look good. If I’m going to find a good partner in the future, I have to look good.”*

### 3.7 Question 15: discussing suggestions from family or friends

#### 3.7.1 Religion

Ideas among relatives about religious cure were mentioned by several participants. This could be seen as thoughtful, especially so when turning to religion was not brought up as an explicit suggestion but rather modelled as a potential resource:

*“I know that my mom prayed a lot for me. Not that I think it helped. [ … ] But I know that my parents care about me enormously.”*

On the other hand, it could also be experienced as annoying and humiliating:

*“And there was actually an ex-partner or an ex-boyfriend who was very … He gave a lot of support and showed a lot of compassion for me and my feelings and thoughts and all that. But there was also a lot of pressure about ‘God will help you’. So I’ve heard a lot of that.”*

*“My mother has mentioned a few times that she spoke to a priest and he told her I’m too far from God and stuff like that. [ … ] Sometimes she’ll say, half-jokingly, but I still think there’s a part of her that believes it: ‘A demon has gotten into my girl, who are you?’, things like that. Because she usually says that I was such as happy little girl, as a kid. [ … ] Perhaps she somehow really thinks that I’m too far away from God or that there’s a demon inside of me or something.”*

#### 3.7.2 Visit country of origin

A couple of participants had relatives who had suggested that visiting their country of origin would bring about much-needed change for them. However, none of them actually ended up travelling there:

*“I knew it would be good. But I’m not sure it’s that good when you’re thinking about suicide and need the kind of healthcare that I can’t get in Nigeria. Where I don’t even really know the language—I know it, but not that well. [ … ] But I was ready to leave. We were checking for tickets and everything. But I think I said to my mom: ‘But what do I do if I become suicidal while I’m there?’ And then she was also kind of scared and it was like: ‘Oh God, you’re right. What do we do then?’”*

#### 3.7.3 Usual “dad wisdom”

Several participants described how parents—typically their fathers—had mostly suggested simplistic solutions, such as “have a banana!”, that showed them that they did not truly comprehend the seriousness of an eating disorder:

*“My dad was like: ‘Well, you just got to eat healthy, exercise, and everything will be fine.’ He still says that. ‘Just eat good, exercise—and if you exercise, eat more.’ [ … ] I think he believes that’s the greatest advice ever. He’s been telling me for a month.”*

### 3.8 Question 16: addressing misunderstandings in the clinical encounter

This question did not elicit many accounts of misunderstandings during the present interview, but clearly prompted participants to discuss previous experiences in specialist eating disorder treatment and other healthcare settings.

#### 3.8.1 Discrimination

A couple of participants described outright discrimination from healthcare providers. For example, one woman had on several occasions been sent home from the emergency ward with what turned out to be serious illness, experiences she ascribed to racial prejudice:

*“And then he was like: ‘Nah, it’s nothing to worry about. I think you’re exaggerating a little bit. [ … ] You just need to rest. It’s all in your head.’ It’s not! I feel bad mentally because I’m in so much pain! And it’s not only me it’s happened to. I don’t know what to do about it. I can’t emphasize my symptoms, because then it’s like: ‘Immigrants, they’re so dramatic!’ And I can’t just tell them how I feel, because then they don’t take me seriously. Even though I’m very good at communicating my needs.”*

One man described how staff would sometimes offer him less support, based on stereotypes of Black men as tough and unemotional:

*“I’m older, I’m from another part of the world, and I don’t always get the same kind of support as other patients. They get more help or [the staff] are more compassionate towards them. Maybe they expect me to be able to handle certain things. [ … ] It might make them perplexed, kind of like my looks don’t match what’s inside of me.”*

#### 3.8.2 Downplaying racism

Some participants described that previous therapists had ignored or downplayed experiences of racism that had affected their psychological well-being. This had prompted them to specifically seek out therapists of color, since they felt that many White therapists did not understand or appreciate the impact of racism:

*“When I talk to friends whose parents are from Nigeria, for them it’s really important to find a therapist who can relate. For me, as long as I get the help I need I don’t care who you are. Sure, I had a period when I was 27 or 28 or something when I felt like: ‘Oh God, Swedish people’—I mean White people—’they just don’t get it.’ So I looked specifically for a Black therapist.”*

#### 3.8.3 Pressure from non-White therapists

There were also a few mentions of how non-White therapists had occasionally made participants feel singled out in treatment. For example, one participant described how a medical doctor had acted as if her migrant background would somehow make it less difficult for her to grapple with an eating disorder:

*“But [my physician] told me when I was there, he just: ‘I’ll be frank with you: the majority of these Swedish girls we see here, they’re not going to get much better. But I believe in you. You feel like the kind of girl who…’ It was like he was sort of ‘behind the scenes’ with me. He felt some type of connection or community with me because I’m also of a foreign background. It was as if I were the first [non-Swedish person] he had seen there.”*

#### 3.8.4 Lack of insight into other cultures

A couple of participants had experienced that previous therapists lacked a general insight into cultural differences:

*“They have a hard time even imagining that somebody else’s life can be different from their own. And of course I’ve encountered that in healthcare too. I had a therapist once who thought that everybody’s lives look the same in all countries. That the languages differ, but that the system is always the same. [ … ] And it was very difficult talking to him, because he took it for granted that everybody else lived the way he did. And he couldn’t even fathom the possibility that they might not.”*

#### 3.8.5 Pressure to break with family

A couple of participants had felt pressured by therapists to rebel against honor-based family culture and break with their families, reflecting a simplistic view of honor culture and an underestimation of the importance of migrant family networks as support systems:

*“The solution has often been oversimplified by therapists. [ … ] The simplify the solution, that it’s supposedly easy to just follow your own path and break [with the family] and move away and all that. That’s how I’ve felt.”*

### 3.9 The CFI debriefing instrument for patients

Quantitative analysis of the participants’ evaluations of the CFI showed that they experienced it as a useful, feasible, and acceptable instrument. For the factor Clinical Utility, ranging from -2 (i.e., least useful) to 2 (i.e., most useful), the mean answer was 1.1 (SD=0.8). For the factor Feasibility, also ranging from -2 (i.e., least feasible) to 2 (i.e., most feasible), the mean answer was 1.7 (SD=0.3). For the factor Acceptability, ranging from 0 (i.e., least acceptable) to 10 (i.e., most acceptable), the mean answer was 8.3 (SD=1.1). No single question was perceived as particularly difficult by any participant in the formal evaluation.

## 4 Discussion

The findings from this study, exploring the role of the CFI as a tool in the assessment of eating disorders in eight adult patients with a migration background, indicate that the CFI can be a feasible, acceptable, and useful instrument in working with this patient group. With the help of the CFI, a number of relevant aspects of the participants’ experiences were identified, including associations between perfectionism and the migrant condition, the importance of religious faith as support in times of distress, and the impact of racism and discrimination. Some of the CFI questions more consistently prompted a discussion of various sociocultural aspects of the participants’ lives that were potentially relevant to assessment and treatment, whereas other questions did not produce any distinct patterns.

Ideas about perfectionism as an important causal factor were evident in the interviews, in response to the CFI question 4. Perfectionistic strivings (i.e., setting high standards for oneself) and perfectionistic concerns (i.e., fear of making mistakes and of negative evaluation) are both well-known risk factors for the development of eating disorders (20), although they can also be described as transdiagnostic factors relevant to a broader range of mental disorders (21). Some participants described how being immersed in a ‘high-performance culture’ at work or in peer groups contributed to feelings of inadequacy. This was also linked to professional identity as an important element of the participants’ cultural identity. It can be noted that athleticism and dieting have become important lifestyle markers of career success in contemporary Sweden as well as in many other parts of the world. Whereas business leaders of the past smoked cigars and played golf, successful professionals of today who wish to display their status participate in ultramarathons, adhere to intricate fasting regimes, and equate overweight in employees with poor self-discipline (22). Importantly, however, our participants also clearly linked striving for perfection with their experiences as migrants or as children of migrants. They described being told from an early age about the many sacrifices that their parents had made in order to be able to migrate to Sweden and start a new life, often including loss of previous social position and downward mobility. In order to make the decision to migrate “worth it”, our participants experienced a strong pressure—in the form of both personal high standards and parental expectations—to succeed academically and to pursue a career in a high-prestige profession. Moreover, in order to achieve this success, many participants felt that they had to work harder than their non-migrant peers, due to a lack of resources or expectations of failure. This element of the migrant experience is certainly not new (23, 24); in this context, however, it means that individuals with a migration background may be even more susceptible to the impact of perfectionism as a risk factor for the development of eating disorders, given the specific associations between migranthood and striving for perfection. Addressing migrant experiences of striving for success may very well prove useful in the treatment of an eating disorder.

Previous Danish research has highlighted the CFI question 8 and the two subsequent questions—focusing on the meaning and impact of cultural background and cultural identity—as particularly abstract and difficult to understand for patients and healthcare providers alike (25). In the present study, all participants also reacted with some perplexity to question 8, asking for it to be repeated or explained in more detail. However, after hearing it twice or having briefly discussed various aspects of cultural identity, they could all produce rich and nuanced accounts without much ado. Question 8, when allowing for clarification and illustration beyond the verbatim content of the CFI, was clearly helpful in elucidating a number of crucial but previously neglected elements of the participants’ lived experience, including the importance of religiosity and faith, existing in a state of “in-betweenness”, and being a victim of racism.

Religion and spirituality has previously been highlighted as a potentially important resource in coping with and recovering from an eating disorder (26). Even so, it is well known that clinicians often feel uncomfortable with discussing religious matters with their patients (27), perhaps especially so in a country such as Sweden that has often been described as highly secular. Our findings point to religion and spirituality as a neglected aspect of patients’ lived reality. Importantly, individuals who consider religiosity a vital aspect of their cultural identity often do not adhere to secular privatized and compartmentalized notions of religion as something that happens only on Fridays, Saturdays, or Sundays, for instance; instead, spirituality is typically viewed as an integral part of what it means to be human, to relate to other people, and to live a healthy life. From this perspective, failing to address religiosity in clinical encounters may signal a lack of interest in the patient and become a barrier for communication and trust building (28). In the present study, those participants who brought up religion as a vital part of their cultural identity hardly subscribed to a distinctly cosmocentric worldview (29) by which gods, spirits, and ancestors take center stage—often, they would explicitly denounce what they saw as an excessive and conservative religiosity of the parental generation in favor of a more personal and open-minded spirituality of their own. Nevertheless, religion was important for them. Addressing religiosity in treatment may improve clinician-patient rapport and prove useful in identifying new avenues for psychosocial support; it could also, as evident from some of the participant narratives, reveal unhelpful ideas about sinfulness, lack of personal agency, etc. that need to discussed and problematized.

Numerous theories accounting for the state of in-betweenness experienced by many migrants and individuals belonging to minority groups exist in the literature, including the “third space” of Homi K Bhabha (30), various takes on “Caribbeanness” and the creole (31–33), the “new ethnicities” theorized by Stuart Hall and others (34, 35), the scholarship on intersectionality initiated by Kimberlé Crenshaw (36), and so on. A feature common to many of these accounts is the emphasis on in-betweenness as something-in-itself, a novel and unique state of being beyond any simplistic ideas of métissage or hybridity as a salad bowl of discrete sociocultural categories. Many of the participants in this study had clearly embraced the fairly recent Swedish word for in-betweenness, “mellanförskap”, and used it to describe what they saw as a vital aspect of their cultural identity. In their narratives, the liminal state of in-betweenness was depicted as a resource, a burden, or a little bit of both—mostly, however, it was described as a fact of life. Sometimes participants saw in-betweenness as a positive resource in their lives, valuing the potential for other ways of relating to the world aside from those of the Swedish majority population or of the parental generation. At other times, it could be a nuisance, such as when having to negotiate between parental expectations and the norms of the Swedish society or when not being regarded as a “real” Nigerian for having grown up in an affluent neighborhood with mostly White peers. Observations such as these are reflected in the literature on experiences of children of immigrants, who may often take upon themselves the task of acting as an informal translator between the parental generation and the host society, being familiar with “both worlds”. This responsibility may be unproblematic for some, but there is also an obvious risk that it may contribute to an ambiguity within the family system that undermines normal parental authority, producing feelings of alienation and incapability in both parties (37).

Another theme that emerged in various ways throughout the interviews were experiences of racism and discrimination. Interpersonal and internalized racism are known to be associated with negative mental health outcomes such as anxiety, depression, psychosis, and substance use (38, 39), possibly mediated though an increase in allostatic load. Moreover, structural racism has profound negative impact on many social determinants of mental health, such as household income, employment, education, housing and food security, neighborhood characteristics, etc. (40). Exposure to racial discrimination has also been shown to contribute to disordered eating behaviors, such as overeating and loss-of-control eating (41). Many of our participants described experiences of racism in the Swedish society as something that had shaped their view of themselves and their cultural identity. Some also described flagrant and potentially fatal examples of discrimination and racism in healthcare, which they had not previously discussed with others than friends and family. Notably, the CFI question 16—addressing misunderstandings in the clinical encounter—clearly prompted participants to discuss previous experiences in healthcare settings, not just the current interview situation. The CFI can evidently be a valuable tool in identifying and addressing experiences of discrimination and racism in healthcare.

Several participants also discussed how honor-based norms and expectations affected them and their mental health. A recent Swedish survey found that 17% of adolescent girls and 8% of adolescent boys in Stockholm live in an honor-based family context (42), subjecting them to psychological, physical, sexual, and economic violence and severely limiting their life choices. Our participants reported that feelings of lack of control in different areas of life, mostly due to honor-based expectations, contributed to disordered eating by strengthening the perception of food as the only available arena for self-determination. An alarming finding was also that disordered eating would be seen as a sign of immaturity by family members, thus becoming yet another reason for removing control from the participants’ lives. However, the theme of honor also emerged in responses to the CFI question 16, where participants described how therapists sometimes had a very limited understanding of honor-based family dynamics, expecting them to be able to easily “break free”. The Swedish survey referenced above clearly points to simplistic stereotypes—such as describing parents as “monsters” or viewing honor-based violence as “inherent” to certain migrant or religious groups—as a major barrier to help seeking for those affected (42). It is evident from our findings that honor-based norms may negatively impact eating behaviors, but therapists must be able to address this without demonizing families or turning to racial prejudice if they wish to create an environment in which patients feel safe to open up about their experiences.

With one exception, there is a notable absence of reflections upon the cultural significance of food and eating from the participant narratives elicited by the CFI. This mirrors what might be described as a lack of interest in the social and cultural aspects of food in much eating disorder research, where eating pathology is typically conceptualized as a maladaptive coping mechanism in response to affect dysregulation (43), similar to self-injurious behaviors or substance use, and where food as culture is merely a curiosity (44). An exception to this is the budding field of research on cultural adaptations of eating disorder treatment (45, 46), which usually involve considerations regarding acculturation, family dynamics, and food as care. It may be noted that when participants were explicitly asked (in a separate, broader interview) about cultural aspects of food and eating in their family or their country of origin, almost all affirmed that this had played a role for them in the development and maintenance of eating pathology. Apparently, the CFI does not adequately capture these cultural aspects in the assessment of disordered eating; if a clinician wishes to explore this theme in more detail, separate questions should be asked.

### 4.1 Trustworthiness and rigor

This study adds to the very scarce literature on utilizing the CFI in the assessment of patients with eating disorders. The author who performed the interviews has long experience of working with the CFI and regularly trains healthcare staff in using the instrument, ensuring close familiarity with the interview situation; however, this also means that the findings cannot be taken as guidance on how to implement the use of the CFI in specialist eating disorder services for staff who are previously unfamiliar with the instrument. A further strength is that much effort was put into identifying and counteracting barriers for participants to feel safe and to be able to provide a full narrative during the interviews, as described in the section on reflexivity above.

There are also limitations that may affect the interpretation of the findings reported here. The study involves relatively few participants and should primarily be viewed as a pilot study rather than as a more definitive exploration of the potential role of the CFI in the assessment of eating disorders. Of course, qualitative research does not require specific sample sizes in order to produce meaningful and useful results (47); even so, we certainly expected to recruit more participants for this study. The fact that we were only able to recruit eight participants over a time period of 18 months reflects a relative absence of patients with a migration background in Swedish specialist eating disorder treatment, despite a substantial burden of disordered eating in this group (8).

### 4.2 Implications for the future

This pilot study indicates that the CFI can be a feasible, acceptable, and useful instrument in working with patients with eating disorders. Considering the rich and clinically meaningful narratives provided by our study participants, it is surprising that there are not more reports of the CFI being utilized in the assessment and treatment of eating disorders. One possible reason is the fact that persons of color and individuals with a migration background are relatively underserved in specialist eating disorder treatment (8, 9), diminishing the perceived need for an instrument that focuses on sociocultural aspects. Importantly, the CFI is not meant to be used solely in situations where the clinician/interviewer and the patient/interviewee appear to differ markedly in terms of cultural background; there may very well be relevant cultural aspects that need to be identified and addressed even when clinicians and patients appear to share the same overt cultural identity (10). Even so, our experience is that the CFI is unfortunately often used mostly with patients that are seen as “different” in terms of ethnicity or nationality, in much the same way that culture is often seen as residing in “the Other”. As described above, the present study was designed as an addendum to a larger ongoing qualitative study about barriers to treatment for eating disorders for individuals with a migration background in Sweden. However, exploring the CFI as a clinical tool in the assessment of patients identifying as belonging to the White Swedish majority population would also be of great interest—this could also potentially contribute to nuancing the picture of the usefulness of the CFI for broader groups.

## Data availability statement

The datasets presented in this article are not readily available for reasons of patient confidentiality. An aggregated and anonymized coded dataset is available from the corresponding author upon reasonable request. Requests to access the datasets should be directed to mattias.strand@ki.se.

## Ethics statement

The studies involving humans were approved by Swedish Ethical Review Authority. The studies were conducted in accordance with the local legislation and institutional requirements. The participants provided their written informed consent to participate in this study.

## Author contributions

MS: Conceptualization, Data curation, Formal analysis, Funding acquisition, Investigation, Methodology, Project administration, Writing – original draft, Writing – review & editing. EW: Conceptualization, Methodology, Project administration, Resources, Writing – review & editing. SB: Conceptualization, Methodology, Resources, Supervision, Writing – review & editing.
